# Immersive Virtual Reality Exergames for Persons Living With Dementia: User-Centered Design Study as a Multistakeholder Team During the COVID-19 Pandemic

**DOI:** 10.2196/29987

**Published:** 2022-01-19

**Authors:** John Muñoz, Samira Mehrabi, Yirou Li, Aysha Basharat, Laura E Middleton, Shi Cao, Michael Barnett-Cowan, Jennifer Boger

**Affiliations:** 1 Department of Systems Design Engineering University of Waterloo Waterloo, ON Canada; 2 Department of Kinesiology and Health Sciences University of Waterloo Waterloo, ON Canada

**Keywords:** virtual reality, exergames, persons living with dementia, physical activity, head mounted displays, participatory design, co-development, gaming, older adults, elderly, design, dementia, VR, user-centered, physical activity, exercise, COVID-19

## Abstract

**Background:**

Advancements in supporting personalized health care and well-being using virtual reality (VR) have created opportunities to use immersive games to support a healthy lifestyle for persons living with dementia and mild cognitive impairment (MCI). Collaboratively designing exercise video games (exergames) as a multistakeholder team is fundamental to creating games that are attractive, effective, and accessible.

**Objective:**

This research extensively explores the use of human-centered design methods that involve persons living with dementia in long-term care facilitates, exercise professionals, content developers, game designers, and researchers in the creation of VR exergames targeting physical activity promotion for persons living with dementia/MCI.

**Methods:**

Conceptualization, collaborative design, and playtesting activities were carried out to design VR exergames to engage persons living with dementia in exercises to promote upper limb flexibility, strength, and aerobic endurance. We involved a total of 7 persons living with dementia/MCI, 5 exercise professionals, 5 community-dwelling older adults, a VR company for content creation, and a multidisciplinary research team with game designers, engineers, and kinesiology experts.

**Results:**

An immersive VR exergame called Seas the Day was jointly designed and developed and it is freely available to be played in state-of-the-art VR headsets (Oculus Quest 1, 2). A model for the triadic interaction (health care institution, industry partner, academia) is also presented to illustrate how different stakeholders contribute to the design of VR exergames that consider/complement complex needs, preferences, and motivators of an underrepresented group of end users.

**Conclusions:**

This study provides evidence that a collaborative multistakeholder design results in more tailored and context-aware VR games for persons living with dementia. The insights and lessons learned from this research can be used by others to co-design games, including remote engagement techniques that were used during the COVID-19 pandemic.

## Introduction

### Background

Technology has a key role to play in supporting people of all abilities’ fundamental rights to inclusion and participation. The essential purpose of technology is enabling people to do things they could not otherwise do. However, applications need to be specifically adapted to the needs and abilities of end users for them to be accessible and meaningful [1]. Top–down approaches where designers independently create technologies can lead to well-intended but poorly suited solutions, especially for older adults (defined here as aged 60+) with cognitive, physical, or sensory impairments, such as people living with mild cognitive impairment (MCI) or dementia [2-4]. To meet the needs of such end user groups, a multistakeholder team has been shown to better understand the experiences, challenges, and adoption of technology from different perspectives (eg, end users, health care professionals, service providers, researchers from multiple disciplines, industry designers/developers, and engineers) to form a holistic view [1]. Combining this diversity of knowledge enables creation of novel solutions that can be effective, usable, and adoptable by the end users [5]. Human-centered design (HCD) is an approach where solutions are created by focusing on understanding the context, needs, behavior, and preferences of the people whom the solution will serve. In HCD, the end users’ needs, system requirements, and technology specifications are defined using data from observations, interviews, and participatory design activities. Participatory design is one of the techniques used in HCD in which end users and other stakeholders are actively involved as partners throughout the design process [4,6].

Adopting participatory and collaborative design approaches in HCD has been shown to result in the development of effective solutions and improve the effectiveness of serious games in promoting healthy lifestyles among the end users [7]. However, many requirements of these approaches are reported as barriers that can be difficult to overcome [8]. For instance, increased time and effort are required to come to a common and shared understanding of the problem and possible solutions [9]. As a result, technology development still predominantly relies on conventional and less participatory approaches that often come at the cost of a greater chance of misalignment of the technology with the intended user population [5]. This is particularly the case when designing solutions for people with complex needs or impairments. For instance, understanding the desires, needs, and abilities of older adults with cognitive impairment can be extremely complex, dynamic, and unpredictable considering the physical and cognitive challenges faced by this population [10]. While there is a general agreement on the importance of having a multistakeholder co-design approach to design, implicit complexities such as different viewpoints (eg, people from different sectors or schools of thought), conflicts of interest (eg, intellectual property of the designed solutions), and access to end users that are willing to participate in the design process limit widespread adoption [11].

As put forward by Dixon and Lazar [12], technology for persons living with dementia should support activities that are meaningful to them in a way that respects and reflects their needs, abilities, and perceptions of personhood. As persons living with dementia have wide and dynamic preferences and abilities that are difficult (sometimes impossible) to simulate or speculate about, it is crucial to have their voice as a central part of the design process of technologies intended for them. While actively involving persons living with dementia in technology development has already started and multiple articles have documented valuable insights [2,3,13], the adoption rate is quite low and appropriate/effective methods for supporting their involvement are still being explored.

This paper presents the participatory cocreation of *Seas the Day*, an exergame (ie, a game that is intended to promote exercise) designed to include persons living with dementia/MCI as core end users. *Seas the Day* uses immersive head-mounted virtual reality (VR) to encourage players to engage in exercises that can be beneficial for their health and well-being. The research described in this paper was guided by the question: “How can human-centered participatory design methods be used to involve multiple stakeholder groups in the collaborative creation of VR exergames to promote physical and mental well-being among persons living with dementia/MCI?”. We present our work as a case study of a collaborative co-design wherein we discuss main considerations, roles, and lessons learned through our process of co-designing VR exergames for persons living with dementia/MCI; this includes strategies that were adopted to steer the design process while being involved in the COVID-19 pandemic, a situation that brings many challenges due to the limited access to the target stakeholder groups. We describe how we tackled previously reported issues in designing games targeting older adults with cognitive impairment, in particular: (1) creating solutions that are designed with and for persons living with dementia/MCI by identifying where exceptional needs (eg, cognitive, physical, sensory impairment, and technology literacy) of this population exist and complementing them [14]; (2) integrating players’ needs and preferences early in the design process [15]; and (3) balancing both attractiveness and effectiveness to create enjoyable and useful immersive experiences [8]. This research highlights specific considerations regarding designing exergames for head-mounted displayed-VR (HMD-VR) technology and specifies how using HMD-VR impacted the design choices we made throughout the iterative and participatory process.

### Supporting Aging in Persons Living With Dementia Using Physical Activity: The Opportunity of Exergames

Dementia is an umbrella term for a number of progressive diseases and disorders, such as Alzheimer disease, Lewy Bodies, and Parkinson disease. Symptoms of dementia involve deterioration in cognitive function including impairment in memory, reasoning skills, and the ability to perform everyday activities as well as changes in behavior and mood [16,17]. MCI is a high-risk state for dementia where individuals experience a decline in cognitive abilities, which is not yet sufficient to hinder functional independence. There is ample evidence on numerous physical and psychological health benefits of regular physical activity participation for older adults [18]. For persons living with dementia or those with MCI in particular, physical activity has been recognized as a practical and side effect–free therapeutic strategy for both mitigating and managing the symptoms of MCI and dementia. Regular participation in physical activity can improve functional performance, mobility, activities of daily living (ADLs) among persons living with dementia and those with MCI, and may have a positive impact on their global cognition and balance [19-21].

While some guidelines recommend that persons living with dementia/MCI exercise at least twice a week [22], other guidelines recommend they participate at the same level of activity as healthy older adults—150 minutes of moderate intensity or 75 minutes of vigorous aerobic physical activity and strength training twice a week [21]. However, despite strong evidence supporting physical and mental benefits of physical activity for persons living with dementia/MCI, physical activity participation and adherence are particularly restricted in this population due to the motor and cognitive changes associated with the condition. Various individual, social, and environmental barriers such as lack of motivation, low levels of self-efficacy, apathy, poor access to exercise opportunities, lack of dementia-appropriate exercise programs or safe and accessible community infrastructure, transportation challenges, and societal stigma have been reported as contributors to sedentary behavior among persons living with dementia/MCI [23-26].

Given the increasing number of dementia cases worldwide (expected to double by 2050) [27] and considering the significant health benefits of regular physical activity, it is imperative to develop innovative and effective strategies to facilitate physical activity participation and maintenance among both healthy older adults and those living with cognitive impairment. Serious or applied games, such as exergames, are one plausible strategy to promote physical activity among older adults by motivating participation through the enjoyment of play [28]. VR exergaming is a novel strategy that can encourage physical activity participation and offer exercise routines that require minimal guidance and supervision from the therapists [29,30]. The multisensory and immersive environment of VR exergames (especially those employing HMD-VR) have been previously employed as a therapeutic tool to promote the health and wellness of older adults and to support rehabilitation [31,32]. Studies that explored VR exergames for older adults have shown positive results and demonstrated that exercising using exergaming systems can benefit motor learning and neural plasticity [33-37]. VR exergaming has been found to be a feasible strategy to complement conventional exercise interventions [32,38].

### Exergaming During the COVID-19 Pandemic

Older adults are among the most vulnerable and profoundly impacted during the COVID-19 outbreak and its physical and mental health impacts [39]. Staying physically active during the COVID-19 pandemic is particularly important for older adults because physical activity is a protective factor against viral infections that can increase the immune response as well as the positive benefits toward supporting overall physical and mental well-being [40,41]. However, with the contact restrictions, isolation measures, and exercise facilities closure in response to the COVID-19 pandemic, older adults are facing restrictions of physical activity behaviors that can lead to short- and long-term adverse health consequences [42,43]. For those living in long-term care (LTC) homes or apartment buildings where going outdoors requires moving through shared spaces, risks for physical inactivity and its various adverse health outcomes are even higher. Reduced social connection and increased feelings of loneliness may also decrease older adults’ motivation for physical activity during the COVID-19 pandemic [44-46].

To mitigate the negative impacts of COVID-19 on the health and well-being of older adults (with and without cognitive impairment), various remote and technological solutions have been suggested, such as [47-50]. Concurrently, literature indicates the growing feasibility of using exergaming strategies to enhance physical activity among older adults during the COVID-19 pandemic [51-54]. For example, VR exergaming has been introduced as a coping strategy to facilitate older adults’ at-home physical activity and enhance favorable health outcomes among this population [55,56]. This can be due to the fact that virtual environments are customizable and can be tailored to the participants’ functional and cognitive abilities, including those with MCI/dementia. For individuals reluctant to participate in exercise, the immersive and interactive environment of VR can provide an engaging, entertaining, and motivational means of exercising and target desired physical activity outcomes through the gameplay [57].

While there is promising potential for VR exergames to support older adults, research on preferences of VR exergames among older adults with various cognitive abilities is limited. Additionally, the availability of custom-made content and easy-to-use VR hardware often limit the technology uptake by older adults [58]. Moreover, public health measures to contain the spread of COVID-19 have made it more challenging to carry out participatory and collaborative cocreation activities with vulnerable end users such older adults and persons living with dementia/MCI during the pandemic. Therefore, despite the growing need and technological advances in VR systems (eg, standalone headsets), the use of VR exergames to promote exercise among older adults is still very limited.

### What We Know About Serious Games and Cocreation for Persons Living With Dementia

In order to provide a comprehensive understanding of what has been done in the field of serious games for persons living with dementia/MCI, we present below a comprehensive review of the literature, specifically literature covering VR technologies. The rise of consumer-level HMDs and accessibility of the content have fostered the creation of literature related to both nonimmersive and immersive VR systems adopted for dementia care. Examples are reflected in the following publications: (1) a review of nonimmersive games and simulations including tools for ADL to “brain” games (eg, games for cognitive training or assessment) [59]; (2) a review on the cost-effectiveness of exergaming interventions and their impact on physical, cognitive, emotional, and social functioning of persons living with dementia/MCI, which revealed that only 3 studies met the inclusion criteria (eg, randomized controlled trials, participants diagnosed with dementia, exergames); (3) a minireview of 10 studies on interactive interventions to promote well-being of persons living with dementia/MCI revealed that virtual experiences were enjoyable for the participants and improved their mood and apathy [60]; and (4) the effectiveness of VR (full-immersive and semi-immersive) for persons living with dementia/MCI was reviewed in a recent meta-analysis of 11 studies concluding that immersive VR is a cost-effective, comprehensive, flexible, and potentially useful tool for patient-centered care [61].

The future of VR technologies to foster interactive and therapeutic experiences for persons living with dementia/MCI presents several challenges that have been repeatedly mentioned in most of the reviews. These challenges can be grouped as follows: (1) the need to establish practical guidelines for VR design and implementation for people with cognitive impairments [61]; (2) the need for less anecdotal, more consistent, and more therapeutically and scientifically valid interventions (eg, randomized controlled trials) to systematically document the effectiveness of VR interventions in persons living with dementia/MCI [62]; (3) the opportunity to include noninvasive measurements (eg, gameplay metrics, physiological signals) and system intelligence (eg, machine learning [63]) to better examine changes, propose novel ways for tracking the VR intervention progress, and better quantify its impact [60]; and (4) the need to improve the rationale behind specific game elements (eg, mechanics, technology) and their impact on the outcome measures (eg, cognitive, physical) in order to better define the goals of the interactive interventions [64]. For instance, there is a need to better define the role of the level of immersion offered by the technology (eg, HMDs, 2D screens) in game user experience variables such as engagement or replayability.

## Methods

### Cocreation of the Seas the Day Exergame

This section describes how we carried out our co-design of the *Seas the Day* VR exergame, including presenting the ideation stages, involvement of stakeholders, and a table summarizing the iterative and collaborative design and development process, which was partially carried out during the COVID-19 pandemic.

Our process of cocreating *Seas the Day* was sparked by conversations with exercise therapists in LTC facilities. They expressed a desire for additional ways to engage persons living with dementia/MCI in exercise routines that are fun, particularly for those who do not enjoy or have trouble participating in group-based exercises. They wanted interventions that were not only interactive and engaging, but also inclusive and informed by the participant’s therapeutic goals. They were also interested in exploring what kinds of objective data could be automatically collected through gameplay to track changes/progress in physical and cognitive function over time.

The multistage collaborative design process that ensued occurred over the span of 15 months and was driven by multidisciplinary researchers, exercise therapists, VR game developers, persons living with dementia/MCI in LTC facilities, and community-dwelling older adults. The process is divided into 2 main stages: (1) ideation and planning and iterative design and (2) development process. A special emphasis has been put in describing the techniques, activities conducted, and people involved during each stage.

### Preliminary Work on VR and Exergames for Persons Living With Dementia/MCI

The design process described in this paper is heavily inspired by previous research conducted before COVID-19 by our research team where a set of VR exergames were prototyped and tested. Initially created as a proof of concept, a set of activities in a virtual farm setting was created to engage persons living with dementia/MCI in the use of HMD-VR [65,66]. Important insights from that study were as follows: (1) end users had positive perceptions of the exergame experience using HMDs and were able to engage in the exercise program, (2) involving health care professionals and persons living with dementia/MIC in the design process was incredibly beneficial to creating usable VR environments for persons living with dementia/MCI, and (3) qualitative and quantitative measures demonstrated comparative results between exercising with the VR exergaming program and conventional human-guided exercises. Results from this pilot study were crucial to define the next steps and to identify opportunities of using HMD-VR technology in promoting physical activity among persons living with dementia/MCI [67]. Furthermore, an analysis of the main strengths, weaknesses, opportunities, and threats (SWOT) was conducted to better shape the next steps of the project (Table 1). This approach is conventionally used among companies for strategy building, marketing, and project planning. A focus of the SWOT analysis was to consider how rapidly evolving VR technology could be used to create a solution that could be adopted by persons living with dementia/MCI in dementia care and by elder care institutions.

**Table 1:** SWOT^a^ analysis of VR^b^ exergames previously developed and piloted [67].

Strengths	Weaknesses	Opportunities	Threats
Demonstrated feasibility of using HMD-VR^c^ in persons living with dementia Successfully scaffolded a human-centered design process with persons living with dementia Included 3 activities placed in different scenarios (diversity) Simplified interaction (guided through voice instructions) Included a calibration process for range of motion	Hard to replicate due to hardware and software limitations Discomfort of sweating while using HMDs More suitable for stretching than conditioning exercises Limited visual aesthetics Interactivity errors that can lead to frustration Can get monotonous if used too frequently Data logging system can be difficult to interpret	Use less cumbersome VR system (eg, standalone rather than desktop) Include engaging game mechanics and integrate gamified activities Simplify and improve data logging Facilitate system calibration Explore metrics to track physical and cognitive performance Include full-body interaction	Difficulty in technology uptake due to system’s complexity and cost Content and platform sustainability Design and development time longer than time for student MASc degree Potential motion sickness for some people Users with hearing or visual impairments might not be able to engage fully in content

^a^SWOT: strengths, weaknesses, opportunities, and threats.

^b^VR: virtual reality.

^c^HMD-VR: head-mounted displayed-virtual reality.

### Ideation and Planning

#### Overview

This stage consisted of a set of activities carried out by the multidisciplinary research team to identify, define, and plan for research approaches and goals, game design concepts, and prototyping methodologies required to create VR exergames using HCD methodologies. The process is described in 2 main stages as follows:

#### Defining Research Approach and Partnering With Industry

The initial research team was formed by academic researchers, including professors and graduate students (master’s, PhD, and postdoctoral) with interests/expertise in engineering, human factors, and assistive technologies (n=3); kinesiology and applied health sciences with a focus on exercise programming and delivery for older adults with and without cognitive impairments (n=4); and a game designer with experience in health care applications (n=1) as well as exercise professionals from a local LTC home (n=5). Among the exercise professionals, kinesiologists and recreational therapists with more than 10 years of experience were engaged and were champions during the design process. The strategy to engage exercise therapists consisted of inviting interested professionals of the LTC homes to be part of the research team, involving them in the decision-making processes, and inviting them to take part in the strategic planning of the participatory design process.

The next step was to find an industry partner interested in conducting participatory game design who had related experience/expertise and know-how in developing custom-made VR content and commercialization in health care. This step was particularly challenging as human-centered and participatory design research with older adults has mostly been conducted in academic settings [11]; this is mainly due to differences in the time frames between academia and industry as well as relative novelty of the technology. Our strategy to find our industry partner consisted of researching local company directories and communities to identify potential candidates. One of the researchers met with company representatives, presented the project vision, and discussed ways to establish collaboration.

**Figure 1:**
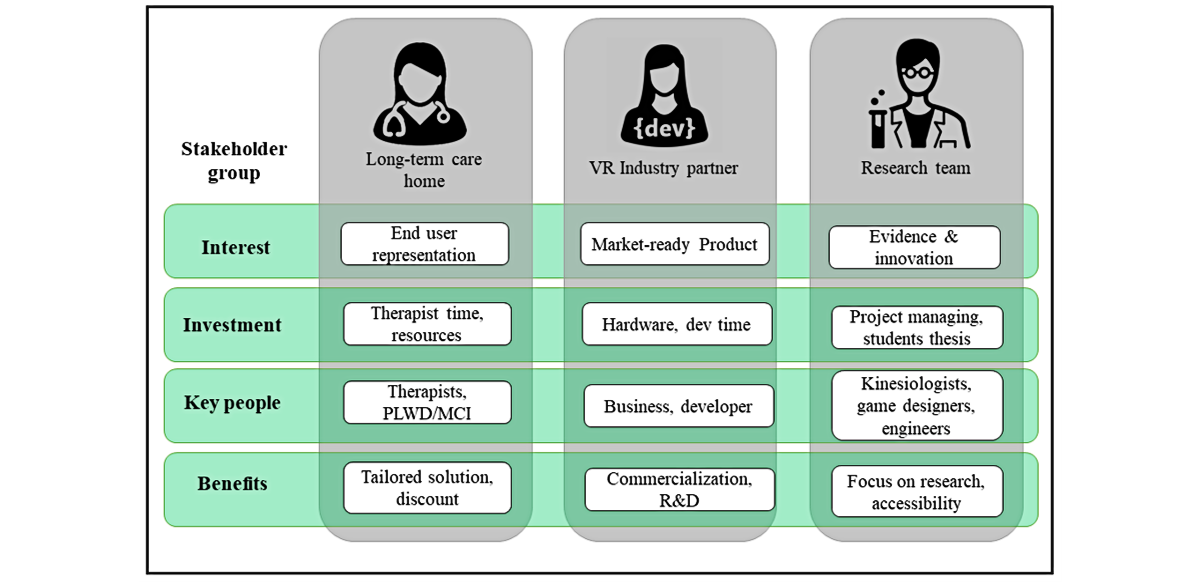
Components of the triadic interaction between the research team, industry partners, and LTC homes. LTC: long-term care; MCI: mild cognitive impairment; PLWD: person living with dementia/MCI; R&D: research and development; VR: virtual reality.

A healthy, robust, and valued partnership is grounded in perceived benefits that are equal to or greater than the investment for each partner. The nature of the stakeholder with their specific interests, key people, required investment (eg, time, activities), and benefits are summarized in Figure 1. The main investment for the LTC facility is represented as the time spent by the exercise professionals to partake in the participatory design process, playtesting sessions, and performance evaluation of persons living with dementia/MCI participating in the design process. Additional resources, rooms in the home to meet about the project, availability of the exercise professionals and other personnel, and use of display devices (eg, projectors or TV screens) for facilitating discussions were part of the LTC stakeholder investment (in-kind contribution). As a benefit, the LTC facility will get a discount on the final product for 5 years and will have a tailored solution that implements needs and ideas from their staff and residents, thus facilitating technology uptake. A shared intellectual property agreement was also negotiated that defined up-front how innovation from the project would be shared in a way that was deemed to be equitable to the researchers, industry partner, and LTC facility.

To facilitate technology deployment, the industry partner provided 2 state-of-the-art, standalone VR equipment (eg, Oculus Quest) for both the research team and the LTC facilities. As part of their in-kind investment, the company allocated a specialized development team to create the virtual environments as well as to conduct research on new gameplay metrics. The company also spearheaded the development of the business model of the envisioned system to provide a sustainable and financially feasible proposal to the team. The benefit for the industry partner is having the exclusivity of commercializing the product as well as gaining experience and insights on working closely with both potential clients and a multidisciplinary research team. By engaging with academia, the industry partner is eligible to receive government grants to support research and tax breaks; our team has taken advantage of both.

Finally, the research team had the mission of carefully planning and managing every step of the process; this included creating activities to appropriately engage all of the stakeholders by designating academic subteams with graduate students, research assistants, and principal investigators aligned to the different research outputs. Having an industry partner facilitated the designing and development of usable and scalable games and allowed the researchers to stay more focused on the scientific aspects of the activities (eg, game user research, evaluation, design of evidence-based games). The involvement of an industry partner also increased the chances of creating immersive exergames that are widely accessible (eg, through purchase, freemium) for both health care and academic settings compared with when no perspective or active stakeholder is involved in commercialization during the design process.

The output of this planning process is a detailed and structured *Work Plan* that is shaped and agreed upon by all stakeholders and that serves as the guide for the design that allows for the creation of the activities, timelines, and milestones for the team.

#### Defining the Game Design Concepts and Requirements for VR Exergaming in Persons Living With Dementia/MCI

##### Purpose

After assembling the multidisciplinary team, a series of design activities (described below) were conducted to explore the different perspectives of the team and to create cohesion, empathy, and mutual understanding. Because some of the team members were not familiar with the symptoms and abilities associated with MCI and dementia, we used existing user personas to describe some of the characteristics as well as the most representative needs and motivations to facilitate the exchange of information among the team members [15,59]. Three researchers who were new to MCI/dementia shadowed 3 exercise sessions in LTC to gain some first-hand experience with exercise programming and delivery for persons living with dementia/MCI in this setting.

##### Physical Activity and Therapeutic Requirements

To narrow down the scope of physical activities to ones that are appropriate for this population (including considering limitations [21,22] and risks of using HMD-VR technology with older adults [68]), we consulted with team members who have expertise in exercise therapy on multiple occasions via dedicated ideation meetings. This resulted in our decision to focus our design on exergames targeting upper limb movements that have been shown to improve endurance, flexibility, and balance. This decision is supported by the results of a systematic review on the effects of exercise on persons living with dementia/MCI in care homes, which reported that exercise intervention that combined aerobic, strengthening, and stretching activities had the greatest benefit [69]. We intentionally designed and developed seated exergames to increase player’s safety and reduce risk of falls [15,70] while simultaneously incorporating guidelines that consider both physical and cognitive capabilities for exergames in persons living with dementia/MCI [71].

A list of desired movements and targeted joints along with their correlation with physical fitness was defined (Table 2), which considers (1) exercise routines carried out in the LTC facilities with persons living with dementia/MCI, (2) recommendations for exercise prescription in older adults [72], and (3) physical challenges specific to persons living with dementia/MCI (eg, on average a decrease in mobility, balance, and strength) [21,69].

**Table 2:** List of movements to be included in the VR^a^ exergames for persons living with dementia/MCI^b^.

Targeted joint/limb	Desired movement(s)	Application for persons living with dementia/MCI
Cervical	Neck flexion and extension (bending the head forward and backward) Neck rotation (turning the head to the left and right)	ROM^c^ Flexibility/Mobility ADL^d^
Shoulder	Shoulder flexion (frontal arm raise) Shoulder abduction and adduction (side arm reach) Shoulder rotation (360° circumduction) Overhead arm stretch	ROM Flexibility/mobility Endurance ADL
Elbow/Wrist	Elbow flexion and extension (biceps curls) Elbow supination and pronation (outward and inward rotation of the forearm) Wrist flexion and extension (tilting toward the palm and tilting toward the back of the hand)	ROM Flexibility/mobility Endurance ADL
Trunk	Trunk flexion and extension (bending forward and backward) Lateral flexion (side bending) Trunk rotation	ROM Flexibility/mobility Weight shifting and postural balance (seated) Core strength ADL

^a^VR: virtual reality.

^b^MCI: mild cognitive impairment.

^c^ROM: range of motion.

^d^ADLs: activities of daily living.

##### Concept Ideation and Brainstorming Activities

Three main activities were conducted to conceptualize the VR exergames: (1) literature review of exergame design frameworks, (2) inclusion of simplified game design elements to improve communication among stakeholders, and (3) brainstorming sessions.

##### Exergaming and Design Frameworks

A literature review was conducted to explore existing design frameworks used to create engaging and effective exergaming experiences [73]. One of the design frameworks specialized in exergaming for healthy lifestyle promotion, called the *dual flow model* [8], was used to guide the design process. In the dual flow model, the design is guided by balancing both effectiveness and attractiveness in exergames to maximize the level of engagement while keeping the beneficial aspect of the games. The implementation of the dual flow model framework was achieved by mapping the 3 main components of an exercise training session (ie, warm-up, conditioning, and cool-down) with individual activities suggested by persons living with dementia/MCI and their therapists [65]. These activities were integrated in each stage of the exercise session (ie, warming up, conditioning, cool-down) using a different game mechanic for each one considering the recommended exercise intensity and duration.

##### One-Page Level Design

**Figure 2:**
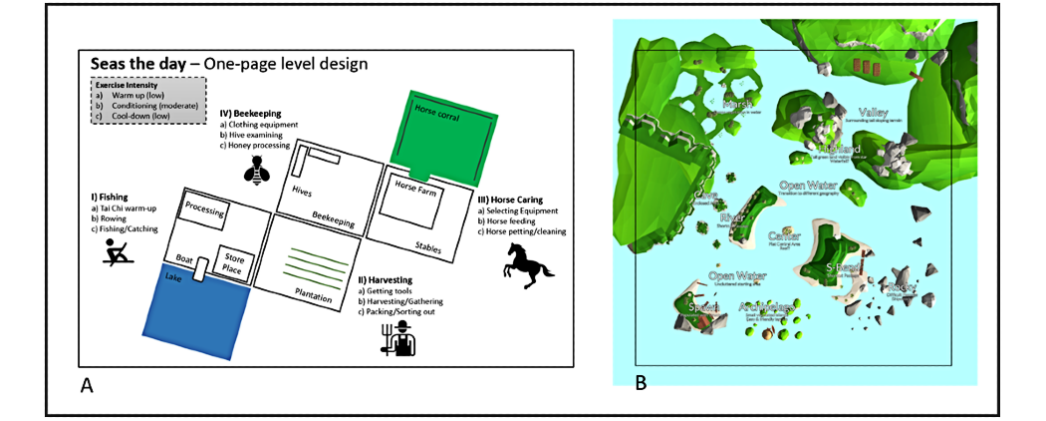
One-page level design of the Exerfarm Valley concept. (A) Four-level design sketch representing the activities and main game levels envisioned (including Seas the Day). (B) Seas the Day level design discussed with the development team of the virtual reality company specifying the spatial characteristic of the elements in the virtual environment.

To facilitate the communication across the research team and stakeholders, a 1-page game level design (Figure 2A) [74] was used where exercise components (eg, stages, intensity) were outlined and 4 different game levels were initially proposed. The farm theme was chosen as suggested by the results of our pilot study and because of its broad acceptability among persons living with dementia/MCI and exercise therapists [66]. The game is named *Exerfarm Valley* and its concept consists of 4 different levels that recreate a productive farm: fishing, harvesting, horse caring, and beekeeping; these activities were chosen from a list of activities generated through discussions with the exercise therapists and researchers and in consideration with their mapping to the movements defined in Table 2. *Exerfarm Valley* was envisioned by the game designers, integrating the input from different stakeholders and considering other similar exergaming approaches found in the literature [15,70]. The initial design was presented and discussed with the research team for further refinement. Each game level has 3 different stages including specific activities that reflect warm-up, conditioning, and cool-down movements. In the iterative design and development stage, details of each game level were discussed and integrated to facilitate a more concrete and streamlined development process. For instance, Figure 2B shows the design of the rowing/fishing level (called *Seas the Day*) after discussing it with the development team.

##### Envisioning and Brainstorming

To further explore the initial design, 2 brainstorming sessions were conducted to create paper prototypes and discuss possible game mechanics and scenarios of the VR exergames.

**Figure 3:**
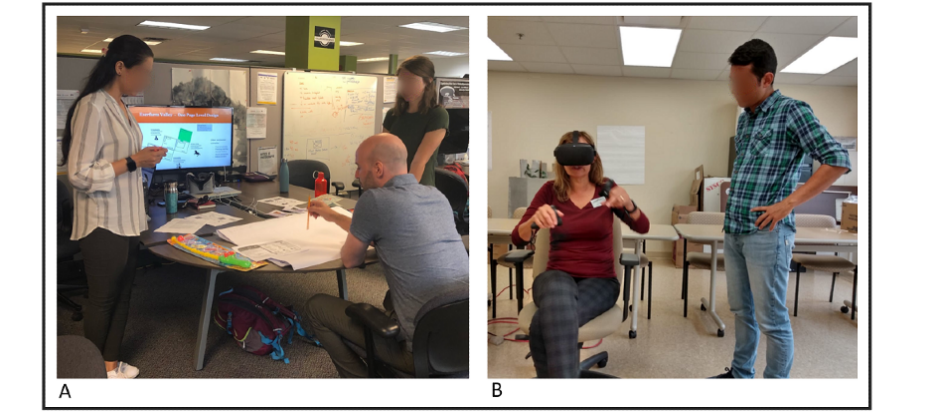
Brainstorming sessions. (A) Introductory session carried out involving multiple stakeholders of the project. (B) Exploratory sessions carried out with exercise therapists to introduce the head-mounted displayed-virtual reality technology and collect ideas about game mechanics.

The *first brainstorming session* (Figure 3A) was carried out with (1) 2 VR specialists (from our industry partner), (2) 3 health care professionals (1 per each institution: university, LTC partner, industry), (3) 2 external researchers with experience in designing digital technologies for persons living with dementia/MCI, and (4) 2 graduate students with experience in user experience design. The goal of the session was to create multidisciplinary subgroups to (1) introduce and expose participants to the different perspectives of the team and (2) collect ideas on the activities and narratives of the envisioned games. The scenario was defined as instructions that were given as follows:

- *Target population:* persons living with dementia/MCI in LTC settings (persons living with dementia/MCI personas were used [75]);
- *Game purpose:* engage players in seated upper limb exercises wearing a standalone VR headset; and
- *Interaction structure:* 15 minutes of exergaming using the VR headset eliciting physical activity responses according to the warm-up, conditioning, and cool-down stages (exertion cards were used to filter the ideas [76]).

The final result encompassed a set of ideas of virtual activities for various game levels (eg, fishing, rowing, animal care). These ideas were used to create a game design document (ie, document detailing the envisioned game specifying game elements such as mechanics and aesthetics) to be used as an input for the VR company to start off with the prototyping process; having the industry partner as an active stakeholder in the brainstorming session was invaluable to building a shared understanding of the opportunities and challenges as well as creating a backdrop with which they could frame ideas.

The *second brainstorming session* (Figure 3B) was conducted in 2 LTC facilities with 11 exercise professionals: (1) 2 physical therapists, (2) 3 kinesiologists, (3) 3 recreational therapists, and (4) 3 kinesiology students. The session aimed at exploring ideas on exercise movements, activities, and game mechanics. The game concept was introduced by defining the farm theme and the 3 intended stages (warm-up, conditioning, cool-down) with their respective timing and intensities. The goal of the session was twofold: (1) to gather therapists’ feedback on the use of the HMD-VR technology in promoting physical activity in persons living with dementia/MCI and (2) to collect ideas on specific activities, feedback modalities, and instructions in each stage. We used a large paper layout dividing the 3 stages and specifying the exercise intensities and asked the therapists to write down or sketch the specific activities they would like to see on each stage, providing as many details as possible. Three researchers analyzed the qualitative results using an affinity diagram approach after moving the concepts to sticky notes and clustering the ideas to build affinity maps [77]. These generated themes centered around (1) the feasibility and requirements of using standalone HMD-VR devices in the LTC (eg, network connectivity, privacy, and adaptation of existing spaces or elements) and (2) specific game mechanics that can be used by persons living with dementia/MCI in the envisioned *Fishing* (Figure 2A) game level (eg, exploring while rowing, fishing with rod, hand-biking, scuba diving, and beach activities such as stretching or playing sports).

### Iterative Design, Development, and Playtesting

An iterative, agile, and cyclical human-centered development process was carried out to merge research concepts, design requirements, and technical feasibility to shape the requirements of the prototype. Each iteration consisted of short prototyping–testing–evaluation cycles, coordinating the research team, exercise therapists, end users, and the development team from our VR partner. Playable prototypes were developed approximately every 2 weeks during a 6-month period. Versions of the games were installed on the VR equipment and then playtested by the research team, end users, or therapists to collect feedback and prepare a playtesting report suggesting required changes and potential improvements for the new prototype. Playtesting sessions were carried out both in-person (before the COVID-19 pandemic) and virtually via audiovisual conference platforms (during the COVID-19 pandemic). While the exergame was initially designed with and for persons living with dementia, because of the limitations of accessing persons living with dementia/MCI in LTC due to the pandemic, we conducted a remote, 1-week pilot with community-dwelling older adult volunteers who agreed to receive the headsets at their home and play our exergame 3 times. The development was initially focused on the creation of experimental game mechanics associated with the movements and therapeutic requirements previously discussed. Other game design elements such as aesthetics and story were gradually added to our exergame, based on feedback from the end users and other stakeholders during the playtesting sessions. In total, 3 playtesting sessions were conducted with 7 persons living with dementia/MCI (6 females; mean age 81.3 years) from 2 LTC homes, a total of 9 exercise providers (both from LTC and community, including the ones in the research team) with experience in dementia care (7 females; mean age 38.1 years), and the researchers to evaluate different aspects of the exergame. Table 3 summarizes the objectives, participants, and insights of each playtesting session. After completing the first playtesting session with persons living with dementia/MCI, the COVID-19 pandemic and public health restrictions forced us to halt in-person research, resulting in access restrictions to persons living with dementia/MCI and exercise providers at LTC homes. Similarly, other preplanned participatory design activities to continue cocreating and playtesting different game design concepts with exercise professionals and persons living with dementia/MCI had to be paused.

**Table 3:** Playtesting sessions.

Playtesting name	Objectives	Participants	Methods	Main insights	
Rowing and desired visual elements (number of sessions: 2)	*Game mechanics:* Rowing forward Rowing backward Rowing turning (left, right) *Game aesthetics:* Desired visual elements *Game technology:* Motion sickness and comfort	7 persons living with dementia 5 exercise therapists (from LTC^a^ facilities)	*Face-to-face playtesting:* 20 minutes interaction Individual, semistructured Overseen by exercise therapists Debrief with the therapists at the end of the session.	All players learned easily how to row in VR^b^ using the prototype Rowing backward/forward and turning left/right were intuitive for most participants When asked about desired visual elements, end users preferred animals (eg, fishes and birds), nature and landscape (eg, sunset, mountains), and other boats and more people. One player could not complete the test because the headset was uncomfortable. Therapists mentioned the importance of adding cues or elements to guide the navigation.	
COVID-19 outbreak					
Rowing improvements and game level design (number of sessions: 1)	*Game mechanics:* Rowing and navigation around the level designed (Figure 3B) *Game aesthetics:* Oars aspect and positioning Game objects and water effects *Game technology:* Capture player’s responses and behaviors	4 community exercise providers with experience in dementia care (who were not working in LTC)	*Remote playtesting:* 1-hour discussion Online (through Zoom) Semistructured focus group	Add configurable menu to define (1) session duration, (2) player’s position calibration to facilitate rowing. Add configurable menu to define (1) session duration, (2) player’s position calibration to facilitate rowing. Modify world physics to have more natural tree shaking and water waving effects. Adding cues to guide participants (eg, signs, audio clips).	
Conditioning and cool-down stages, rowing and fishing integration (number of sessions: 3)	*Game mechanics:* Rowing and fishing cohesion Dolphin as exercise intensity modulator *Game technology:* Player’s responses and behaviors Game aesthetic, story Narrative to aid engagement	Research team (without LTC exercise therapists)	*Remote playtesting:* 1-hour discussion online (through Zoom)	Add strategies to avoid getting stuck while rowing. Better define the virtual world limits by adding buoys regarding the dolphin: (1) sounds should be added to facilitate prompting, (2) dolphin’s behavior should help in meeting exercise intensities in conditioning. Variables such as attention paid to the animals in the scenario as well as the response of players to haptic stimulus can be used to quantify reaction time. Consider movement limitations when fishing to avoid persons living with dementia from getting frustrated.	

^a^LTC: long-term care.

^b^VR: virtual reality.

## Results

### Seas the Day: Final Game and System Overview (Description)

**Figure 4:**
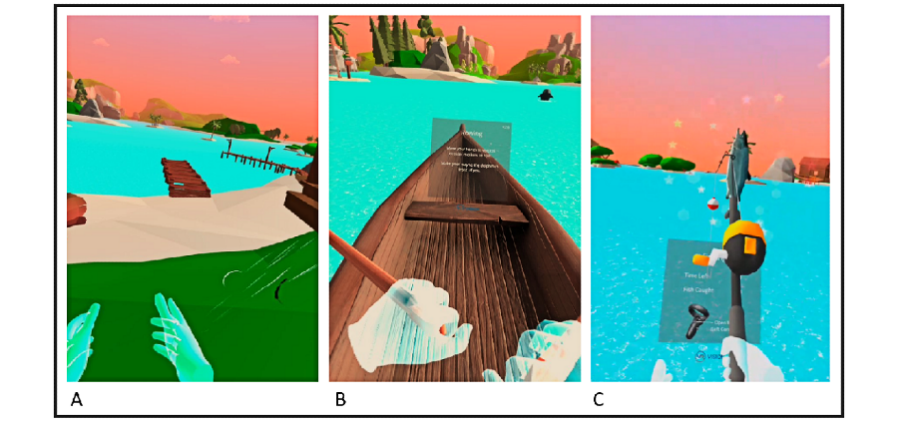
Seas the day screenshots showing the game levels of Tai Chi (warm up, left), rowing (conditioning, middle), and fishing (cool-down, right).

*Seas the Day* is an immersive HMD-VR experience that transports seated players to a virtual seaside with different activities that encourage exercise movements that have been shown to be beneficial to persons living with dementia/MCI [21,69]. *Seas the Day* places the players in a tropical environment surrounded by animals, hills, and water. Three activities lasting a total of 15 minutes were created (Figure 4 and Textbox 1) to align with the design requirements and needs identified in conceptualization and playtesting sessions.

**Activities to align with design requirements and needs identified in conceptualization and playtesting sessions.**

*Tai Chi (warm-up, 3 minutes)*

A set on a beach with a sunset in the horizon and birds seen and heard in the virtual scene. Players start the experience with a short Tai Chi routine that encourages upper limb movements that are connected with the virtual scene (Figure 4A). To guide players in performing the correct movements, a leaf-shifting metaphor is used wherein players are instructed to hold a floating leaf with 2 hands and guide it through trajectory paths drawn up in front of the players. Examples of the incorporated Tai Chi movements for range of motion are “wings of a bird” (moving arms to the side followed by a folding-like movement of the arms toward the chest), “open the door” (raising arms straight up and bring down with elbows bent) and “flower shifting with the breeze” (hands moving side to side).

*Rowing (conditioning, 9 minutes)*

The activity invites players to explore the tropical environment while rowing in a boat using 2 wooden oars attached to their virtual hands. This stage has been created as a conditioning phase as the rowing game mechanic involves exercising the muscle groups described in Table 2 aiming to improve strength and aerobic fitness (Figure 4B). As shown in Figure 4C, this game level has different spaces to explore such as a marsh, small archipelagos, and a valley and rocky spots among others. Hanging bridges, waterfalls, and thatched cottages are some of the objects that players will see while rowing. Five different animals have been included based on the suggestions collected in the playtesting sessions: fish, dolphins, dogs, cows, rabbits, and birds. To guide players, 2 aids have been included: (1) a virtual dolphin that swims specific paths and encourages players to follow along and to explore the virtual environment while keeping an adequate pace, and (2) voice-over narrations recorded by an experienced exercise therapist aiming to guide players on the movements and activities to perform. Following the dolphin is optional and, if players do not wish to do so, they will still be able to explore the environment at their own pace and preference. In this way, players are encouraged to keep exploring the environment by rowing the boat while multiple stimuli appear at different times and places to make the conditioning exercise more enjoyable. Figure 4B shows a screenshot of the rowing activity with the virtual boat, virtual hands, the environment, and the dolphin.

*Fishing (cooldown, 3 minutes)*

The activity comes after rowing, where players are transported to a fishing scene where they are encouraged to fish using a rod attached to their virtual hands. Neck rotations, elbow flexion, and elbow extensions are the main movements included in this game mechanic (Figure 4C). Fish are placed in the visual periphery (180°) and jump intermittently. The players are asked to use one of their hands to throw the fishing line to a targeted spot. Once in the water, the bait is ready for the fish to take; when one does so, the controller will start vibrating and players have to “pull” the rod out to hook the fish. Once hooked, players have to carry out a series of 6-10 repetitions of the desired movement (elbow flexion–extension) to get the fish and put it inside a bucket in the boat. By bringing the fishing rod close to the other hand, the players will be able to switch the hand used to hold the rod and repeat the fishing process. Figure 4C shows a screenshot of the moment where the player catches a fish and brings it to the boat. The game rewards the fishing efforts by displaying the fish in front of the player and moving it to the bucket.

### Main Characteristics

Key features of *Seas the Day* are presented in Textbox 2.

**Key features of *Seas the Day*.**

*Three exercise stages with 3 game mechanics*

*Seas the Day* has been specifically designed to align with exercise recommendations for persons living with dementia/mild cognitive impairment (MCI) by creating an experience that is accessible and autonomously adjusts the exercise intensity for appropriate warm-up, conditioning, and cooldown stages of the exercise training. This is achieved by proposing different game mechanics (ie, Tai Chi, rowing, fishing) for each subgame that are suitable for persons living with dementia/MCI using data from playtesting sessions along with exercise professionals’ recommendations. Game mechanics were designed to be intuitive, realistic, and adapted to the older population (ie, no buttons required, no teleportation) without frustrating those with limited mobility, as suggested by guidelines for exergames for persons living with dementia/MCI [71]. The main goal was for the game mechanics to achieve a balance between the serious intent of the game while keeping the fun and joy of playing in an immersive environment [74].

*Narrative guiding players through the activities*

From the playtesting sessions and previous research [67], we noticed that guiding players in performing targeted activities was a key component to create an effective gameplay experience and to elicit the desired physical responses. Thus, a game narrative approach was integrated into *Seas the Day* by using voice clips recorded by an exercise therapist within the research team with expertise guiding persons living with dementia through exercise therapy. Narrative has been found to be an experience booster in both exergaming and virtual reality (VR) experiences that enhances engagement [78], positively impacts perception of physical activity [79], and reduces motion sickness [80]. For instance, the following script was used as a voice over to encourage players to initiate and guide rowing: “There is nothing better than a trip in a rowboat! You’re holding the oars for the boat in your hands. Can you see the dolphin? I think she wants to play...let’s try to follow her!”

*Automatic data logging*

*Seas the Day* includes a data logging system that automatically captures movement and game variables during the gameplay. After each session, the system creates a data file containing the time series reflecting the changes of the game variables for the session and stores it in the headset internal memory. Examples of game events being recorded are (1) Tai Chi (time that players were following the path, time to complete each exercise); (2) Rowing (boat speed, distance traveled, number of strokes [total, right, and left oar], boat collisions, dolphin status [eg, away, following], birds approaching the boat, and time for the player to respond to it); and (3) Fishing (number of fish caught, pulling the rod up [repetitions], fish taking the bait, and time for the player to respond). As capturing physical and cognitive performance of players was a feature frequently discussed among exercise therapists and the research team, Seas the Day includes experimental variables in all the game levels that aim to capture (1) head and upper limb range of motion of players using kinematic variables (eg, position and acceleration from headset and controllers) and (2) reaction time using game events associated with cognitive function (eg, birds approaching/landing on the boat, pull out the controller after vibration when fishing).

*Configurable options to customize the exercise session*

To facilitate the customization of the experience, a configuration menu was integrated where therapists can modify components of the exercise session: (1) *duration* of 10, 15, or 20 minutes was given for each session; the distribution of time for each exercise stage is automatically configured and (2) *calibration* of the position in the boat for rowing with adjustable height of the avatar’s position to ensure that rowing can be performed smoothly. Novel standalone VR systems (as the Oculus Quest 2) also have improved the calibration by including inside-out tracking technology (the cameras are in the headset and therefore, the calibration of the player position is automatic).

*Seas the Day* is freely available to be played using the Oculus Quest 1 and 2 headsets and can be found at Reality Well platform [4] and at the Oculus digital store [81]. The game complements a suite of VR content (360 videos, 3D tours, and interactive games) created by our VR industry partner and is (to the authors’ knowledge) the first effort toward creating content through a participatory design process (involving various stakeholders such as the company, research team, persons living with dementia/MCI, and exercise therapists) and making the game available to anyone who wishes to use it.

### Pilot of At-Home System Deployment With Community-Dwelling Older Adult Volunteers

Because of subsequent access restrictions to our target population (persons living with dementia/MCI) due to the COVID-19 pandemic, we decided to carry out a final pilot playtesting and co-design session with community-dwelling older adults. The goal of this pilot was to gather feedback regarding the exergame and overall user experience as well as to co-develop a workable protocol for remote deployment in the community of the system, using the exergame, and remote physical and cognitive assessments to quantify potential outcomes of playing our VR exergame (Multimedia Appendix 1). The resulting protocol will be used in our future feasibility study, which aims to remotely deploy the exergame with 20 community-dwelling older adults.

The study protocol, including safety and hygiene procedures for using HMD-VR devices safely at home and within the COVID-19 context, was approved by the University Ethics Board (Multimedia Appendix 2). We then shipped the equipment to our community-dwelling older adult test group members; shipping was done via a prepaid courier service. Five community-dwelling older adults were added as test group members, and the remote activities in Textbox 3 were carried out.

Upon completion of the pilot playtesting and remote assessment sessions, our older adult team members indicated several important aspects related to their experiences and opportunities for improvement, which are summarized in the 4 categories in Textbox 4.

**Remote activities for the community-dwelling older adults.**

*Week 1: Introduction to virtual reality and conducting assessments*

An Oculus Quest 2 virtual reality (VR) headset with the *Seas the Day* exergame was shipped to the participants along with a custom-designed, printed manual to facilitate the technology uptake at home. Remote sessions with a research assistant were also conducted on how to use the system and play the games. A battery of assessments were conducted during Week 1 to estimate cognitive and physical abilities: (1) *Cognitive function* (Montreal Cognitive Assessment, Verbal Fluency Test, Oral Trail Making Test, Flanker Test, 4 perceptual tasks [response time, simultaneity judgment, sound-induced flash illusion, temporal order judgment]); (2) *Mental well-being* (Geriatric Depression Scale and Physical Activity Affect Scale); and (3) *Physical activity* (Physical Activity Scale in Elderly, Exercise Self-efficacy).

*Week 2: Playing Seas the Day*

Older adult test group members were asked to play the exergames 3 times during Week 2 at their convenience with a maximum of 1 time per day. They were asked to play the game for 15-20 minutes while seated, and when finished playing, rate their level of perceived physical exertion and enjoyment. We also asked them to make notes regarding their observations and thoughts about the VR exergaming experience.

*Week 3: Debrief with the research team*

Two activities were conducted during this week: (1) a 30-minute long semistructured interview with each older adult, and (2) a 90-minute long focus group with all 5 older adults, members of the research team, and a member from our industry partner. The purpose of conducting interviews and the focus group session was to better understand experiences and to collaboratively figure out how to improve the protocol.

**Aspects related to experiences of older adult team members and opportunities for improvement.**

*Study process and remote support*

The older adults in the test group enjoyed the social aspect of high levels of interaction with the researchers but felt communication through email was overwhelming. They stated the Week 1 process and assessments needed to be simplified to avoid confusion, such as not finding the correct links or having difficulties to complete the tasks. Test group members also wanted to know more about the purpose of assessments, their test scores, and final results of the future study. They suggested having more introductory sessions with the virtual reality (VR) equipment to facilitate the use of the system (eg, calibration, content selection, exiting the game) and improve rapport with the research team. Overall, the experience of receiving and shipping back the headset and printed material was satisfactory.

*Exergaming experience and playability*

Overall, playing the exergame was perceived as a positive experience and the virtual environment produced a pleasant and engaging exercise experience for the test group. They reported that the most challenging part of the game was when they were asked to row while following a virtual dolphin. The rowing mechanics were perceived to be unrealistic for people with previous rowing experience, although it was easy to follow for other users. The members of the test group found the game easy to play, and the voice and sound effects were considered to be relaxing. Playing the game was perceived as a light-intensity physical activity for all 5 test group members; some suggested making the gameplay more challenging and others wished there were more diverse activities to do. Finally, some participants indicated interest in knowing more about the exergaming design and development process and how our study results may impact future development of the games.

*VR technology experience*

The experience of using the VR hardware for at-home exercise was generally positive but still challenging for most participants. While the instructions in the custom-made VR manual were found to be sufficient (although somewhat lengthy), issues related to calibration, buttons, and locating the controllers in the physical environment after wearing the headset were mentioned during the interviews and focus group sessions. Some participants also reported the need to ask for family members technology support while playing (eg, for troubleshooting or calibration). Launching and exiting the game were also challenges among test group members who were interacting with a VR system for the first time. None of 5 community-dwelling older adults in the test group reported motion sickness or feeling disorientated after the sessions. However, during the introductory session, 1 of the older adults in the test group had difficulties to launch the game and, while troubleshooting with the research team, she spent more than 30 minutes using the VR and reported feeling nauseous.

*Remote assessment*

The test group found performing the online assessments challenging. The computer-based tasks were perceived as being monotonous and repetitive, which resulted in test group members feeling bored or overwhelmed by the amount of time spent in the assessments. A more integrated accompaniment of researchers was suggested to better support participants when completing the online tasks. There was a disparity in technology that was used and some test group members reported having difficulties in completing the tasks when using certain devices (eg, touch computers with small keyboards).

## Discussion

### Principal Findings

Our research assembled a collaboration of disparate professionals and people with lived experience to form a cohesive, productive, and focused multidisciplinary team to create engaging and tailored exergames that can promote physical activity for older adults, including persons living with dementia/MCI. While most research is conducted under specific circumstances (including adhering to COVID-19 restrictions), a key contribution of our work is to demonstrate a working model that blends the interests, investments, key stakeholders, and benefits of an interaction among the academics and private institutions that develop dementia-centered solutions with the potential of being adopted in LTCs as well as generates a sustainable business model for the private sector. The second key contribution of this research is establishing and describing a process, including the purpose, inputs, methods, and outputs of each stage, in a way that others can adopt and adapt it. Providing details regarding the HCD-based digital games for persons living with dementia/MCI is crucial if we are to learn from each other, which in turn will result in appropriate, viable, and replicable methodologies [32]. Clear methodology and careful processes for collaborating with persons living with dementia/MCI are particularly important to ensure their needs and perspectives are considered in the conceptualization, design, and development. While our process still has room for improvement, this research provides a real-world scenario, exposes major challenges, and highlights important design aspects that should be considered when creating immersive games for persons living with dementia/MCI.

While serious games for persons living with dementia/MCI offer much promise, they are far from realizing their full potential. The time, money, and other resources required to design, develop, and implement a VR system must be clearly offset by the benefits for the end users. To achieve this, more efforts must be made by the content creators toward defining, refining, and implementing strategies that support the inclusion of persons living with dementia/MCI and their care partners in the creating process. Accessible solutions should reflect the abilities, values, and needs of the end users to support the uptake and enhance sustainable use of the technology.

### Lessons Learned Through the Creation and Piloting of Seas the Day

#### Leave the Laboratory to Create a Cohesive and Complementary Team

We challenge researchers to leave their laboratories and seek out industry and other nonacademic partners. These partners substantially contribute to the design, scalability of the solution, and enable the team to take a larger, systems-based approach. Connecting with and involving partners from the beginning enabled us to collaboratively define project scope, methods, and outcomes that complemented and benefited all partners. This approach was crucial to building trust, motivating engagement, and creating shared feelings of success when milestones were achieved. In our case, our industry partner goals are well-aligned with our project, this is, they have complementary leadership (eg, the Vice President is an experienced nurse), and have a genuine interest in making a positive change in the lives of persons living with dementia/MCI. This synergy helped to provide momentum through roadblocks. Our LTC partner was keen about the project from the onset. They supported the project by approving their therapists to be core members of the research and offering to pay for backfill (eg, someone to do the therapists’ job while they were working on our project). This resulted in therapists as key team members who guided the process, including facilitating access to LTC, learning about exercise with older adults, and access to persons living with dementia/MCI, all of which have been found to be significant barriers to the development of supportive technologies for persons living with dementia/MCI [14].

#### Now Is the Time to Develop Usable VR for Persons Living With Dementia/MCI

The popularity of VR and its potential to be adopted during and after the COVID-19 pandemic are unprecedented (eg, telemedicine [82]). The pandemic has significant adverse effects on the well-being of persons living with dementia/MCI in LTC homes. These challenges should be quickly and efficiently addressed. VR is well positioned to mitigate some of these challenges; however, there is much work to be done to realize this opportunity. First, the design of custom-made solutions using VR that can be safely implemented in LTC homes during COVID-19 requires close collaboration with therapists and staff, who are finding their time more limited during COVID-19 because of work demands. Second, playtesting with end users (and especially persons living with dementia/MCI) is very challenging or impossible because, at best, they need support from the therapists to start using the system and, at worst, are simply unavailable for research because of COVID-19 restrictions. In our process, we relied on iterative objective (eg, data recorded from the system) and subjective (eg, opinions and observations) feedback from exercise therapists and persons living with dementia/MCI who playtested to guide our design process. In their absence, we have been relying on recordings that team members watch remotely when in-person playtesting is not an option; however, this is significantly slower and less informative.

#### Therapists Are Problem Solvers and Game Designers by Nature

Therapists are constantly looking for new approaches to engage persons living with dementia/MCI in different therapeutic and leisure activities (usually on a tight budget and a busy schedule). They are accustomed to thinking outside the box to develop novel solutions to difficult problems. For instance, the idea of using an animal character to guide a “tour” around the tropical environment to modulate exercise intensity came from a therapist when we asked: “How do you think we can make the rowing activity more fun and engaging for persons living with dementia/MCI?”. As guided by the therapists, we are exploring novel ways of visualizing data to provide therapists and their clients with objective measures of exercise (to be presented in a forthcoming paper). This aspect is challenging as it is a blend of system capabilities and information that results in new forms of data that are readily understood by therapists. To design this, therapists need to envision how the gaming system could be used to augment and improve the conventional methods as well as what information they were not currently working with but would be helpful to have. In our case, inviting therapists as members of our research team created a sense of belonging, long-term commitment, and ownership of the project, which allowed everyone to feel more comfortable, honest, and direct when discussing ideas or exchanging opinions. In short, having therapists as co-designers of our exergame was a very fruitful and enriching experience.

### Limitations Found When Older Adults Use VR Technology

In addition to the lessons learned, limitations presented through the use of VR technology must be addressed. The *weight* and *cost of the headsets* have continuously decreased over the last decades, making VR headsets increasingly accessible and comfortable. Further, limitations arise from the physical hardware of headset itself, as HMDs can be bulky both in size and in weight. Improper fitting of HMDs can cause further discomfort and strain on the neck muscles and indeed feelings of discomfort and dislike of wearing a headset have been previously reported in the literature [83]. We have used Oculus Quest 1 and 2, which weigh approximately 500 g, and have provided participants with a detailed manual as well as one-on-one support with the setup process prior to and during the data collection to help minimize strain and correct placement of the headset.

*Technology know-how*, ranging from limited to no previous experience to expert users [84-86], is a barrier for uptake of VR in older adults as well as other populations. Once a user has gained access to the device, further limitations may appear. For example, to have agency and to be able to explore a virtual environment, the user must be able to use hand controllers to interact with their surroundings. As previous research has found that hand controllers can be complicated for some older adults, especially those living with MCI or dementia, this can limit their interaction with the environment [67]. Therefore, the use of haptic gloves or other controller-free interfaces has been recommended as they may be more intuitive to use [83,87]. Indeed, our playtesting sessions with community-dwelling older adults indicated some issues when using the controllers.

*Cybersickness* is also a concern, with symptoms of nausea, sweating, salivation, apathy, headache, abdominal discomfort, disorientation, postural instability, oculomotor disturbance, and eyestrain being the most commonly reported [88-91]. In VR, the dynamic environments are designed to induce a high degree of immersion enabling an illusory perception of self-motion, known as vection. However, because a user is usually stationary (eg, standing of sitting), the vestibular and proprioceptive organs receive minimal afferent input which can cause sensory conflict, leading participants to experience cybersickness. Given that cybersickness has been associated with detriments in user performance, safety, immersion, presence, and acceptance [39,88,91-94], it is necessary to examine how this may impact the participants who partake in our exergame intervention. Although our pilot project did not lead to any reports of cybersickness, we were provided with feedback regarding the directionality of rowing being incongruent with reality, which may lead to future participants experiencing cybersickness.

Finally, there is a *risk of injury* from one’s external environment when using an immersive HMD virtual environment [95-97]. During this period, users of VR have limited, if any, visibility of the real world as well as limited real-world aural stimulation as many virtual environments include visual and sound cues that are designed to be immersive and distract attention away from the real world. These factors can lead to collisions with real-world objects that can cause injury. Suggestions to mitigate such an outcome range from engaging in VR in a safe area with protected railings to sitting while playing [95]. As such, Seas the Day has been designed to be played while seated and while remaining within the guardian setup.

### Limitations and Future Work

Our process has several limitations. While the research was designed to include end users in several playtesting sessions throughout the project, the COVID-19 outbreak critically limited our access to both persons living with dementia/MCI and exercise professionals working with this population. Collecting information from a homogeneous group of fragile older adults during a global pandemic situation involves multiple challenges such as (1) the constantly changing regulations and governmental policies in developed countries and (2) the individual measures adopted by LTC homes to protect the residents. The recruitment of persons living with dementia/MCI was carried out by exercise professionals in the LTC homes, whereas the recruitment of the community-dwelling older adults for the pilot was by a snowball sampling method. Both recruitment processes have limitations related to the little control researchers have over the participants and their demographics as well as the potential lack of representativeness of the recruited participants. Besides, due to the qualitative nature of the HCD process, the sample size is normally low due to the in-depth analysis required to create engaging game mechanics using playtesting methods and comprehensive player-centric models.

Nevertheless, the feedback collected during the playtesting sessions carried out before the COVID-19 pandemic allowed collecting rich information on player’s preferences regarding visuals, interaction issues in the virtual environment, and perceptions about the use of VR technology. Additionally, we were able to engage community exercise professionals who had a history of working in LTC homes. We also managed to engage community-dwelling older adults to co-develop the deployment protocol, which was invaluable in helping us to refine the protocol for deploying the system and related testing for at-home exercise training programs. Our HCD process did not intend to create generalizable models (eg, user personas, empathy maps) of persons living with dementia/MCI [3] and their preferences for playing VR games. We intended to collect usable information that could inform our game design process and provide insights into which game elements were more suitable to elicit the required movements (Table 2) while keeping the fun of exergaming.

As the game was initially created to be played by persons living with dementia/MCI, some community-dwelling older adults found the game was not very challenging, especially as they became more accustomed to it. While there is nothing impeding healthy older adults to get benefits from playing *Seas the Day*, the challenges and game design process were tailored to those with cognitive impairment, therefore it is not surprising that healthy older adults may find the game and game activities easy to accomplish. Therefore, generatability of the findings should be carefully considered because we have playtested the game with 2 groups of very different older adults (community-dwelling and persons living with dementia/MCI). This research represents a first stage in this project. Future work includes evaluating the effectiveness of the VR system in 2 populations: persons living with dementia/MCI in LTC assisted by exercise therapists (once COVID-19 allows it) and community dwelling-older adults at home.

We will also continue to develop the technology; the next stage of our research will focus on the development of intelligent algorithms to create adaptive gaming experiences that include physiological (eg, cardiovascular [98]) and kinematic (eg, motor control [99]) data to modulate gameplay. In addition, our game development will focus on creating new game scenarios to provide a more diverse, multithematic, and enjoyable virtual farming experience.

### Conclusions

This research provides insights into how HCD can be used to actively involve multiple stakeholders (including end users, researchers, and industry partners) in designing and developing VR exergames that are tailored to older adults with cognitive impairment. The results of our process demonstrate a replicable model of interaction that blends the needs and preferences of end users with those of exercise providers. The value of including end users and exercise therapists’ feedback throughout the game design process results in an enriched game design with elements familiar to and preferred by end users that are potentially effective in eliciting desired exercise movements that produce measurable health outcomes. Also, the inclusion of an appropriate industry partner that specializes in VR content development was crucial to producing a set of exergames with characteristics that are closer to a finalized product (Multimedia Appendix 3).

## Appendix

Multimedia Appendix 1 VR equipment sanitizing protocol.

Multimedia Appendix 2 Cognitive and physical assessments.

Multimedia Appendix 3 Seas the day is an interactive experience created to foster wellbeing in persons living with dementia using virtual reality. A collaborative design process involving exercise professionals, persons living with dementia, kinesiologists, the VR Vision design and development team and researchers in human factors from the University of Waterloo.

## Abbreviations

ADLs: activities of daily living
HCD: human-centered design
HMD-VR: head-mounted displayed-virtual reality
LTC: long-term care
MCI: mild cognitive impairment
ROM: range of motion
SWOT: strengths, weaknesses, opportunities, and threats
VR: virtual reality
