# Supplementary material for: Immersive Virtual Reality Exergames for Persons Living With Dementia: User-Centered Design Study as a Multistakeholder Team During the COVID-19 Pandemic
Source: JMIR Serious Games. 2022 Jan 19;10(1):e29987. doi: 10.2196/29987 (PMC8772876; doi:10.2196/29987)
Supplement: Multimedia Appendix 2 [file games_v10i1e29987_app2.pdf]

ASSESSMENTS

Montreal Cognitive Assessment (MoCA)

Design and Procedure

MoCA will be administered as a screening tool for cognitive impairment (though potential participants will not be excluded based on their MoCA scores). The MoCA has high sensitivity and specificity for detecting mild cognitive impairment (MCI) and dementia. This will be used as an additional descriptive measure of the participants' baseline cognitive health, in addition to the general questions about physical and mental health (from the demographics questionnaire).
The MoCA is not and will not be used for diagnostic purposes, however, it will be used by the researchers to gain insight regarding the sample's baseline cognition and to confirm participant's eligibility in the study (a MoCA score of < 13 suggests severe cognitive impairment). MoCA will be administered remotely and by one of the research team members. Prior to its administration, participants will be informed that this test is solely for research purposes and will not be used as a capacity instrument to determine their abilities in activities such as driving.

Get Active Questionnaire (GAQ)

Design and Procedure

GAQ, developed by the Canadian Society for Exercise Physiology (CSEP), will be used to screen-in the participants as they enter the study to ensure their safe participation in physical activity and exercise. GAQ is self-administrated and includes the following sections: i) Prepare to become more physically active; ii) Assess current physical activity; iii) General advice for becoming more active, and iv) Declaration. In the first section, four questions are asked about diagnosed medical conditions, symptoms, and any restrictions to physical activity. The second section includes a feature unique to pre-participation screening and the third and fourth sections provide general advice for becoming more active and a declaration.

Physical Activity Scale for the Elderly (PASE)

Design and Procedure

PASE will be used to assess the participants' physical activity behavior. Participants will be asked to self-report the frequency they participate in leisure activities (e.g., outdoor walking, light, moderate, and strenuous sport and recreation, and muscle strengthening) by indicating never, 1-2 days/week (seldom), 3-4 days/week (sometimes), or 5-7 days/week (often). Activity duration will be indicated as either less than 1 hour, between 1-2 hours, 2-4 hours, or more than 4 hours.

Geriatric Depression Scale Short Form (GDS-15)

Design and Procedure

GDS-15 (15 point version) is a depression assessment tool specifically designed for older people. Similar to MoCA, GDS-15 will be used solely as a screening tool (and not for diagnostic purposes) GDS will be self-administrated with the purpose of researchers gaining insight regarding the sample's baseline depressive symptoms. Potential participants will not be excluded based on their scores. GDS comprises 15 questions about how the participant has felt over the past week. Questions require yes/no answers.

Trail Making Test (TMT)

Design and Procedure

TMT is a neuropsychological test and can reflect a wide variety of cognitive processes. TMT will be delivered using an online platform. The test is consisted of at least two conditions: condition A, the participant is instructed to draw lines to connect circled numbers on a sheet of paper or computer screen in a numerical sequence (i.e., 1-2-3, etc.) as rapidly as possible while still maintaining accuracy, Condition B, the participant is to draw lines to connect circled numbers and letters in an alternating numeric and alphabetic sequence (i.e., 1-A-2-B, etc.) as rapidly as possible. The goal of the test is for the participant is to finish both parts as quickly as possible, and the time taken to complete the test will be used as the primary performance metric.

Verbal Fluency (VF) Test

Design and Procedure

VF is a widely used test of executive function.VF will be delivered using an online platform and participants will be given 60 seconds to produce a list of animal names. This test will be performed to explore how VR exergames can potentially impact verbal fluency and verbal learning in community-dwelling older adults.

Flanker Task

Design and Procedure

Flanker task includes a set of response inhibition tests used to assess the ability to suppress responses that are inappropriate in a particular context. Similar to other cognitive tests, Flanker will be delivered using an online platform. There will be three types of stimuli: Incongruent, congruent, and neutral stimuli represented by arrows/letters.

- Congruent stimulus- Flanker items call for the same response as the target and may appear identical.
- Incongruent stimulus- Flanker items call for the opposite response of the target and are represented by different symbols.
- Neutral stimulus- Flanker items neither call for the same response nor evoke response conflict

This is an example of what a participant may see in a Flanker Task

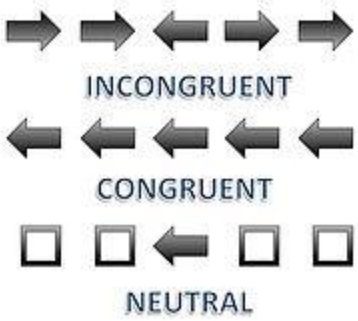

Physical Activity Affect Scale (PAAS)

Design and Procedure

The PAAS is self-administrated and will be used to evaluate affective states among participants. Affective states are thought to be highly influenced by exercise, thus leading to the development of the PAAS as a concise affect measurement tool. The PAAS has twelve items, which can be further broken down into four sub-domains each further broken down into three mood states positive affect (upbeat, energetic, and enthusiastic), negative affect (miserable, discouraged, and crummy), physical exhaustion (tired, worn-out, and fatigued) and tranquility (calm, peaceful, and relaxed).

Usability and Game User Experience Questionnaire

Design and Procedure

This is a self administrated questionnaire developed based on validated usability and acceptability models (e.g., SUS, TAM, SNs, etc.) in order to explore users' perceived usefulness, perceived ease of use, perceived enjoyment and intention to use after interacting with the VR exergame system. Participants will be asked to rank each question from 1 to 5 based on how much they agree with the statement they are reading. 5 means they agree completely, 1 means they disagree completely.

Exercise Self-Efficacy Scale

Design and Procedure

This scale is self-administrated and will be used as a predictor of the adoption and maintenance of exercise behavior. Also, it will be used to assess the participant's beliefs in their ability to continue exercising in the future. This scale includes 9 statements and participants will rank each statement from 1 to 4 based on how sure they are: 1 means very sure and 4 means not sure at all.

Sound induced Flash Illusion (Si

FI)

Materials and Stimuli

Participants will be asked to sit in a quiet room while directly facing their personal computing device. Participants will be asked to place their personal computing device at arm's length, equating to an approximate distance of 57 cm, and the visual stim will be presented in the form of white circles, subtending 2° of visual angle. The visual stimulus will appear 8° below the fixation cross (0.5^o visual angle, appears at the center of the screen and will remain on display throughout the trial) for 16 ms. The auditory stimulus will be presented in the form of a beep (3500 Hz, 16 ms, 68 dBA) through speakers [AB1] either connected to the participant's device or external speakers placed besides the screen. In order to reduce temporal predictability, each trial began with the stimulus being presented after a delay of 1000-3000 ms. Psychopy will be used to generate the stimuli. A computer keyboard will be utilized by participants to input their responses for each trial. As seen in previous research (Chan et al., 2015), participants have reported having seen or heard three or more stimuli, thus responses will not be limited to '1' or '2', as participants can perceive more than the presented stimuli (audio or visual). The entire experiment will be self-paced with no emphasis on speed of responses.

Design and Procedure

There will be three conditions (vision-only, auditory-only, and audiovisual). In the vision-only block, one or two flashes will be presented, and the participants' task will be to indicate how many flashes were presented. In the auditory-only block, one or two beeps will be presented, and the participants will be asked to indicate how many beeps they heard. The following SOAs that will be used in these conditions will be as follows: 0 ms (one stimulus event), 50 ms, 90 ms, 162 ms, 292 ms, and 525 ms for both 2 beep and 2 flashes conditions (these SOAs were chosen as they cluster around 0, and were generated to follow a logarithmic growth of 0.8 with 50 ms as the anchor). There will be 110 trials in each of the unimodal conditions, where 60 trials for 1 stimulus presentation (0 ms) and 50 trials for the two-stimulus presentation, with an equal number divided between the SOA conditions (10 trials for each SOA condition).

The audiovisual trials will consist of two control conditions (1 beep/1 flash and 2 beeps/2 flashes) and the illusion condition (2 beeps/1 flash). In the audiovisual control conditions, the auditory and visual stimuli will be presented simultaneously. In the 2 beeps/1 flash (illusory condition) auditory-lead trials, the auditory stimulus will be presented first, after which the auditory and visual stimuli will be presented simultaneously following a variable SOA. In the 2 beeps/1 flash vision-lead trials, the first auditory stimulus will be accompanied by a visual stimulus and the second auditory beep will be presented following a variable SOA. The following SOAs will be utilized for this study: ±50 ms, ±90 ms, ±162 ms, ±292 ms, and ±525 ms; here '+' indicate vision-lead trials while '-' indicates auditory lead trials. In the 2 beeps/2 flashes trials, the same SOAs will be used. These conditions will be randomly presented within the testing block to avoid response bias. Participants will be asked to fixate on fixation cross for the duration of the task and report the number of flashes seen. Participants will be asked to ignore the auditory stimuli and to respond as accurately as possible. All conditions will be repeated 10 times for a total of 150 trials. Practice trials will also be presented in order to help familiarize the participants with the task.

Simultaneity Judgment (SJ) task

Materials and Stimuli

Participants will be asked to sit in a quiet room while directly facing their personal computing device. Participants will be asked to place their personal computing device at arm's length, equating to an approximate distance of 57 cm, and the visual stim will be presented in the form of white circles, subtending 2° of visual angle. The visual stimulus will appear 8° below the fixation cross (0.5^{o} visual angle, appears at the center of the screen and will remain on display throughout the trial) for 16 ms. The auditory stimulus will be presented in the form of a beep (3500 Hz, 16 ms, 68 dBA) through speakers either connected to the participant's device or external speakers placed besides the screen. In order to reduce temporal predictability, each trial began with the stimulus being presented after a delay of 1000-3000 ms. Psychopy will be used to generate the stimuli. A computer keyboard will be utilized by participants to input their responses for each trial where participants will press '1' if they perceive the auditory and visual stimuli to be simultaneous and '2' if they perceive the auditory and visual stimuli to be non-simultaneous. The entire experiment will be self-paced with no emphasis on speed of responses.

Design and Procedure

Participants will be presented with auditory beeps and visual flashes of light on a personal computing device. The visual stimuli will be presented below a fixation cross while the auditory stimuli will be presented through speakers, either connected to the participant's device or external speakers placed beside the screen. Over the course of the task, participants will be asked to indicate whether they perceived the auditory and visual stimuli to be occurring 'simultaneously' or 'non-simultaneously' by pressing '1' (simultaneous) or '2' (non- simultaneous) or in their keyboard to indicate their responses. The following SOAs will be used for this task: 0 ms , ±50 ms, ±90 ms,

±162 ms, ±292 ms, and ±525 ms. During this task they will be presented with 130 trials, as well as practice trials in order to help familiarize the participants with the task.

Temporal Order Judgment (TOJ) task

Materials and Stimuli

Participants will be asked to sit in a quiet room while directly facing their personal computing device. Participants will be asked to place their personal computing device at arm's length, equating to an approximate distance of 57 cm, and the visual stim will be presented in the form of white circles, subtending 2º of visual angle. The visual stimulus will appear 8º below the fixation cross (0.5º visual angle, appears at the center of the screen and will remain on display throughout the trial) for 16 ms. The auditory stimulus will be presented in the form of a beep (3500 Hz, 16 ms, 71.7 dB) through speakers either connected to the participant's device or external speakers placed besides the screen. In order to reduce temporal predictability, each trial began with the stimulus being presented after a delay of 1000-3000 ms Psychpo will be used to generate the stimuli. A computer keyboard will be utilized by participants to input their responses for each trial where participants will press '1' if they perceive the light first and '2' if they perceive the sound first. The entire experiment will be self-paced with no emphasis on speed of responses.

Design and Procedure

Participants will be presented with auditory beeps and visual flashes of light on a personal computing device. The visual stimuli will be presented below a fixation cross while the auditory stimuli will be presented through speakers, either connected to the participant's device or external speakers placed beside the screen. Over the course of the task, participants will be asked to indicate whether the "light came first" or "sound came first" by pressing '1' (light first) or '2' (sound first) on their keyboard to indicate their responses. The following SOAs will be used for this task: 0 ms, ±50 ms, ±90 ms, ±162 ms, ±292 ms, and ±525 ms. During this task they will be presented with 130 trials, as well as practice trials in order to help familiarize the participants with the task.

Note that the stimuli are exactly the same for the SJ and TOJ task and the only difference is the question that the participants are being asked.

Response Time (RT) task

Materials and Stimuli

Participants will be asked to sit in a quiet room while directly facing their personal computing device. Participants will be asked to place their personal computing device at arm's length, equating to an approximate distance of 57 cm, and the visual stim will be presented in the form of

white circles, subtending 2º of visual angle. The visual stimulus will appear 8º below the fixation cross (0.5º visual angle, appears at the center of the screen and will remain on display throughout the trial) for 16 ms. The auditory stimulus will be presented in the form of a beep (3500 Hz, 16 ms, 71.7 dB) through speakers either connected to the participant's device or external speakers placed besides the screen. In order to reduce temporal predictability, each trial began with the stimulus being presented after a delay of 1000-3000 ms Psychpo will be used to generate the stimuli. A computer keyboard will be utilized by participants to input their responses for each trial where participants will press the spacebar as soon as they detect any of the stimuli (light, sound, or light and sound).

Design and Procedure

Participants will be presented with auditory and visual stimuli either simultaneously or each stimulus will appear individually. The auditory stimuli will be presented through speakers, either connected to the participant's device or external speakers placed beside the screen, while the visual stimuli will appear below a fixation cross on the participants' personal computing device. The participants will use their keyboards by pressing the spacebar to indicate perception of the stimuli, regardless of whether "auditory stimulus only", "visual stimulus only", or "if both auditory and visual stimuli" were presented. 300 trials will be presented during this task and will consist of 100 unimodal auditory stimuli, 100 unimodal visual stimuli only, 100 bimodal auditory and visual stimuli, and 6 practice trials to help familiarize the participants with the task. Practice trials will also be presented in order to help familiarize the participants with the task.
